# Effect of music-based movement therapy on the freezing of gait in patients with Parkinson’s disease: A randomized controlled trial

**DOI:** 10.3389/fnagi.2022.924784

**Published:** 2022-10-19

**Authors:** Kun-peng Li, Zeng-qiao Zhang, Zong-lei Zhou, Jian-qing Su, Xian-hua Wu, Bo-han Shi, Jian-guang Xu

**Affiliations:** ^1^School of Rehabilitation Science, Shanghai University of Traditional Chinese Medicine, Shanghai, China; ^2^Yueyang Hospital of Integrated Traditional Chinese and Western Medicine, Shanghai University of Traditional Chinese Medicine, Shanghai, China; ^3^School of Public Health, Fudan University, Shanghai, China; ^4^Department of Neurorehabilitation, The Second Rehabilitation Hospital of Shanghai, Shanghai, China; ^5^Changqiao Community Health Service Centre, Shanghai, China; ^6^Ministry of Education, Engineering Research Center of Traditional Chinese Medicine Intelligent Rehabilitation, Shanghai, China

**Keywords:** Parkinson’s disease, freezing of gait, music-based movement therapy, gait disorders, randomized controlled trial

## Abstract

**Background:**

Progression of freezing of gait (FOG), a common pathological gait in Parkinson’s disease (PD), has been shown to be an important risk factor for falls, loss of independent living ability, and reduced quality of life. However, previous evidence indicated poor efficacy of medicine and surgery in treating FOG in patients with PD. Music-based movement therapy (MMT), which entails listening to music while exercising, has been proposed as a treatment to improve patients’ motor function, emotions, and physiological activity. In recent years, MMT has been widely used to treat movement disorders in neurological diseases with promising results. Results from our earlier pilot study revealed that MMT could relieve FOG and improve the quality of life for patients with PD.

**Objective:**

To explore the effect of MMT on FOG in patients with PD.

**Materials and methods:**

This was a prospective, evaluator-blinded, randomized controlled study. A total of 81 participants were randomly divided into music-based movement therapy group (MMT, *n* = 27), exercise therapy group (ET, *n* = 27), and control group (*n* = 27). Participants in the MMT group were treated with MMT five times (1 h at a time) every week for 4 weeks. Subjects in the ET group were intervened in the same way as the MMT group, but without music. Routine rehabilitation treatment was performed on participants in all groups. The primary outcome was the change of FOG in patients with PD. Secondary evaluation indicators included FOG-Questionnaire (FOG-Q) and the comprehensive motor function.

**Results:**

After 4 weeks of intervention, the double support time, the cadence, the max flexion of knee in stance, the max hip extension, the flexion moment of knee in stance, the comprehensive motor function (UPDRS Part III gait-related items total score, arising from chair, freezing of gait, postural stability, posture, MDS-UPDRS Part II gait-related items total score, getting out of bed/a car/deep chair, walking and balance, freezing), and the FOG-Q in the MMT group were lower than that in the control group and ET group (*p* < 0.05). The gait velocity, the max ankle dorsiflexion in stance, ankle range of motion (ROM) during push-off, ankle ROM over gait cycle, the knee ROM over gait cycle, and the max extensor moment in stance (ankle, knee) in the MMT group were higher than that in the control group and ET group (*p* < 0.05). However, no significant difference was reported between the control group and ET group (*p* > 0.05). The stride length and hip ROM over gait cycle in the MMT group were higher than that in the control group (*p* < 0.05), and the max knee extension in stance in the MMT group was lower than that in the control group (*p* < 0.05). Nevertheless, there was no significant difference between the ET group and MMT group (*p* > 0.05) or control group (*p* > 0.05).

**Conclusion:**

MMT improved gait disorders in PD patients with FOG, thereby improving their comprehensive motor function.

## Background

Parkinson’s disease (PD), a neurodegenerative disorder among the elderly population, was first described by James Parkinson in 1817 (Parkinson, 2002). The prevalence of PD is estimated as 0.3% in the general population and up to 1% for those aged over 60 years around the world (Balestrino and Schapira, 2020). Movement disorders (bradykinesia, resting tremor, rigidity, and postural instability) and non-motor symptoms (constipation, hyposmia, depression, and cognitive impairment) are frequently observed in patients with PD (Nemade et al., 2021). The incidence of PD exhibits a gradually increasing trend, making it urgent to find a treatment that could prevent or delay the progression of the disease (Tysnes and Storstein, 2017; Abbas et al., 2018).

Freezing of gait (FOG) is a serious gait disturbance that affected mobility and increased the risk of falling in people with PD in the middle and late stages (Gao et al., 2020). Patients with PD described FOG as feeling their feet were stuck to the ground when initiating gait, turning, or negotiating obstacles (Moore et al., 2020). Also, FOG contributes to the occurrence of disability, falls, and diminished mobility in patients with PD (Mazzetta et al., 2019). Therefore, it is critical to control the symptoms, reduce functional disability, and improve patient quality of life while treating PD with FOG. Previous studies have offered a number of hypotheses concerning pathological mechanisms, which could be summarized as the following four models: the threshold model (Plotnik et al., 2012), the interference model (Lewis and Barker, 2009), the cognitive model (Vandenbossche et al., 2012), and the decoupling model (Jacobs et al., 2009). Although these models and hypotheses have explained the development of FOG from different perspectives, they are incomplete and only partially explain the pathophysiology and clinical phenotype of FOG. Treatments used for the improvements of FOG warrant further exploration due to its complex mechanism, which involves many neural pathways and transmitter systems. In fact, the responsiveness of FOG to pharmaceutical treatment was limited and not always promising (Espay et al., 2012; Perez-Lloret et al., 2014; Nonnekes et al., 2020). Despite of benefits of symptom management, medication regimens do not necessarily improve gait disorders, like shuffling, step irregularity, freezing, and postural instability. The episodic, heterogeneous nature, and limited responsiveness of FOG to treatment make disease management difficult (Capecci et al., 2016). Thus, we hold a putative concept that long-term drug therapy might only play an adjunctive role in PD with FOG.

Music-based movement therapy (MMT) has become an effective tool to compensate for the indispensability of conventional treatment. Music therapy might be effective for treating damaged brains (Altenmüller et al., 2012; Moore et al., 2014). The goal of music therapy is to induce dramatic changes in brain function to systematically modified behavior (Wang and Agius, 2018). Rhythm, as well as melody, plays a crucial role during music training (Karpati et al., 2017). Humans have the ability to synchronize their movements to auditory beats or rhythms spontaneously (Wright and Elliott, 2014). Cumulative evidence has demonstrated that music-based stimulation training could improve the gait of patients with PD (Ghetti et al., 2017; Ghai et al., 2018; Park et al., 2020, 2021). For a number of brain regions, neurons can respond to brief stimuli by spiking for a long period (Cui and Strowbridge, 2018). Repeated stimulation would strengthen the connection between the two neurons by causing biochemical and anatomical changes (Szelenberger et al., 2020). Hence, music could not only relax the brain but also link brain regions (Yang et al., 2012).

For its positive stimulatory effects on a range of brain regions, music therapy has been used to improve socialization and cognitive, emotional, and motor functions (Gramaglia et al., 2019). A systematic review showed that MMT is an effective treatment approach for improving motor function, balance, freezing of gait, walking velocity, and mental health for patients with PD (Zhou et al., 2021). Combining music therapy and physical therapies, MMT inherits the strengths of music therapy and physical therapies, indicating a more effective complementary treatment compared to traditional exercise and music therapy. With the advantages of simple operation and cost-save, MMT harbors potential research value and a wide range of application possibilities (de Dreu et al., 2012). By far, varieties of diseases have been found to be positively influenced by MMT. Patients could benefit from MMT in terms of their daily activities and motor function, as well as their quality of life. According to previous work, the combination of dance and music therapy improved walking function and the capacity of posture transition for PD (Pereira et al., 2019; Dos Santos Delabary et al., 2020). However, there is considerable research still recommending monotherapy (receiving music therapy or physical therapy only) for PD patients with FOG. In our prior trials, MMT improved limb function in patients with neurological disorders (stroke, spinal cord injury, PD). To sum up, MMT might have potential applications for PD with FOG. A further RCT of high quality was required for gaining further robust evidence for consideration. In the current study, we performed a prospective, randomized controlled trial to further evaluate the efficacy of MMT in PD patients with FOG.

## Materials and methods

### Study design

As a prospective randomized controlled trial, it complied with the Consolidated Standards of Reporting Trials recommendations and was approved by the institutional review board and the Ethical Committee (2018-01). We registered the clinical trial on the Chinese clinical trial registry (ChiCTR1900026063) prior to the first participant enrollment.

### Participants

Participants were recruited from Shanghai’s second rehabilitation hospital through the web platform, outpatient, and poster advertisements at inpatient clinics.

#### Eligibility criteria

##### Inclusion criteria

Eligible participants should fulfill the following criteria (Tang et al., 2019):

①192 Classified as Parkinson’s disease (Daniel and Lees, 1993);

② Absence of organic disease;

③ Can stand at least 30 min without walking aids;

④ Aged 40–90 years and could walk independently for at least 3 meters;

⑤ Suffering FOG, with stage 2 or 3 Hoehn and Yahr;

⑥ Had a good to excellent response to L-dopa or other dopaminergic agents;

⑦ At a stable stage of disease and can communicate normally;

⑧ Provided full informed consent.

##### Exclusion criteria

Those participants meeting the following were excluded from the study:

① Secondary Parkinson’s disease;

② No-response or intolerant to previous treatment;

③ Participated in a clinical trial related to PD in the last 3 months;

④ Received additional treatments.

### Randomization and masking

#### Sequence generation

Randomization was administered by a trained research assistant *via* a random number generator function using Microsoft Excel (version 2016).

#### Concealment mechanism

After the informed consent was signed, the eligible participants were randomly assigned into three groups. This operation was conducted randomly, ensuring optimum allocation concealment. Random numbers used for participant grouping were placed into sequentially numbered, sealed, and opaque envelopes.

#### Blinding

Blinded outcome assessment and data analyses were implemented. The collected data were coded and kept using a computer after removing unnecessary information. Unblinding will not be permissible throughout the trial.

### Study interventions

All interventions were carried out by 10 skilled therapists with over a decade of clinical experience. The detailed interventions were described as follows.

### Music-based movement therapy group

Intervention arm was provided with the MMT besides usual rehabilitation care. Regular exercise was modulated with respect to the musical tempo. There were five training sessions per week, with each session of 30 min.

#### Music selection

Before intervention, therapists selected the music with the following consideration: music preferences and personality, musical parameters such as rhythm, melody, harmony, dynamics, and timbre. Also, music with lyrics was not suitable because it may distract participants. Thereafter, therapists made a customized music playlist, which was loaded into a music player for each subject. In addition, participants had a choice of earplugs or headphones to achieve maximum comfort. The music was played repeatedly in order of the playlist, with a 2-min interval between music.

#### Exercise therapy

Participants underwent exercise training on a trail, which was 5 meters long and 1 meter wide (60 min at a time). Exercises, which were performed to the beat of the music, consisted of flat start walking, turning, narrow space walking, and step training. The participant took the first step with the toes as high as possible, then stepped forward with the heel touching the ground first and waited until the body was fully shifted to the foot before taking the next step. At the same time, swing the arms as far as possible. The participant first stepped with the foot on the same side as the direction of the turn, and then slowly shifted the body in place with small steps and angles as the body turned. After the direction was reversed, participants were instructed to take a step forward. Exercise intensity was judged by whether participants achieved a self-perceived exertion rating of 12–13 (easy) (Scharhag-Rosenberger et al., 2015). All subjects were asked to take a rest for about 10 min in case of fatigue during exercise training.

### Exercise therapy group

Participants in this group were subjected to exercise therapy without music. Patients, wearing earphones but without music, were required to perform flat walking, turning, narrow space walking, and step training five times (1 h each time) every week for 4 weeks.

### Control group

All participants were guaranteed conventional treatment including usual medical care and rehabilitation, comprising the following: ① 192 basic drug treatment; ② 193 physical factor treatment; and ③ 194 daily life ability training. These treatments were performed five times (1 h each time) every week for 4 weeks.

### Outcome measures

Clinical outcomes were evaluated by observers blinded to treatment allocation.

#### Primary outcome measurement

We adopted a three-dimensional gait analysis system to obtain gait information, including gait cycle (double support), kinematic parameters (stride length, gait velocity, cadence), joint angle parameters (ankle: max dorsiflexion instance, range of motion (ROM) during push-off, ROM over gait cycle; knee: max flexion instance, max extension in stance, ROM over gait cycle; hip: max hip extension, ROM over gait cycle), and kinetic parameters (ankle: max extensor moment in stance, knee: max extension moment in stance, max flexion moment in stance).

The 3D motion capture hardware equipment included 12 infrared light-sensitive cameras (Motion Analysis, Rohnert Park, CA, USA), 4 Bertec 3D force measurement tables, 80 A/D channel digital-to-analog converters (National Measuring Instruments, Austin, TX, USA), and 2 HD high-speed cameras. Software programs consisted of Cortex 1.1.0 data acquisition software and Orthotrack 6.6.1 gait data analysis software (Motion Analysis Corp., Rohnert Park, CA, USA). After applying the infrared light-sensitive marker ball to the corresponding body surface position, the participant was instructed to walk about 15 meters in the test area in a usual posture and speed, so that they could complete the adaptation training to the field-testing environment and the state of wearing the fluorescent ball. For static data collection, participants were instructed to stand naturally in the designated position with bilateral upper limbs held laterally for about 5 s. For dynamic data collection, participants were instructed to walk naturally on a dynamometer-lined walkway after the removal of the fluorescent balls from the bilateral medial ankles and medial femoral skeleton. Participants’ gait data for a 5-meter walking distance needed to be collected in at least three sets. For each set, at least three whole gait cycles of the unilateral lower extremity were performed, and real-time kinematic and kinetic data for each joint of the lower extremity were collected. The 3D modeling analysis of the data was performed using the data acquisition software (Cortex 1.1.0) and the gait cycle was defined, processed, and analyzed using the gait analysis software (Motion Analysis Corp., Rohnert Park, CA, USA) to calculate the spatio-temporal parameters, kinematic and kinetic parameters of each joint of the lower limb.

#### Secondary outcome measures

The secondary evaluation indices included the evaluation of the comprehensive motor function and FOG-Questionnaire (FOG-Q) (Tambasco et al., 2015). We employed the Unified Parkinson’s Disease Rating Scale Part II and III (UPDRS II, III) gait-related items scores to assess the motor function, while the FOG-Q was used to assess FOG.

### Statistical analyses

This was a randomized controlled trial, aimed at assessing the efficacy of three methods in the management of PD with FOG. The key observation index was gait parameters. As no studies performed the three-dimensional gait analyses to appraise the effect of MMT on FOG, we adopted results of the unified Parkinson’s disease assessment scale (UPDRS III) from our exploratory trial for calculations. According to the pilot study, the mean value of UPDRS (III) score contraction in three groups after treatment was anticipated to be 20.7 ± 16.6, 7.9 ± 6.3, and 2.6 ± 2.2, respectively. We fulfilled a two-sided test, with α and assurance (test efficiency) of 0.05 and 90%, respectively. The sample size was calculated to be 66 cases using PASS software (version 15). Further, we calculated an overall sample size of no less than 84 cases enrolled assuming a dropout rate of 20%. However, only 81 patients completed the whole trial finally due to the influence of COVID-19.

Demographic characteristics of patients were described using descriptive statistics. When continuous variables conformed to normal distribution and chi-square, one-way ANOVA was used for comparison among the three groups, and multiple comparisons were performed using Bonferroni’s adjustment method. For categorical variables, the chi-square test or Fisher’s exact test was used. The rank sum test was used if data did not conform to normal distribution. P-values less than 0.05 represented a statistical significance. Notably, to avoid type I errors when making multiple comparisons, adjusted *p*-values were calculated as the *p*-value of 0.05 divided by the number of comparisons. When the *p*-value was less than the corrected *p*-, statistical significance was present.

## Results

### Study participants

**FIGURE 1:**
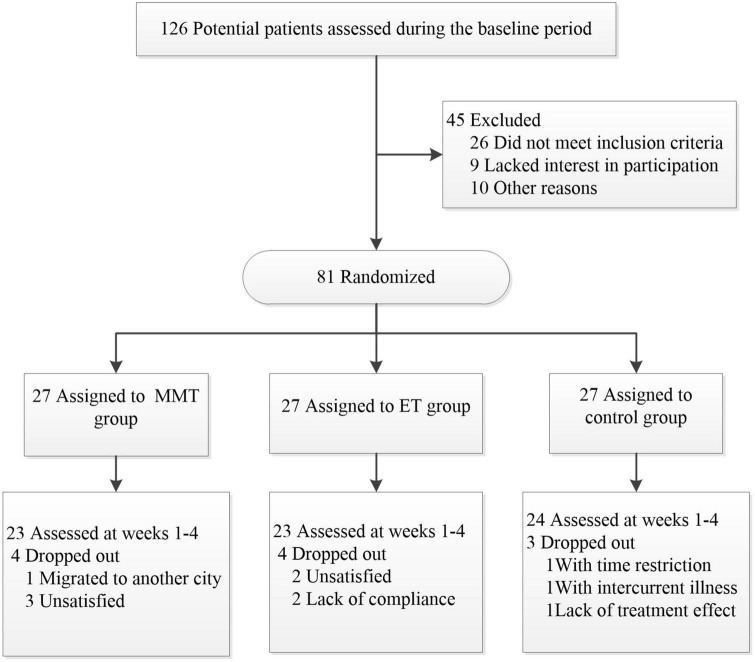
Flowchart of the screening, enrollment, and randomization.

Of these 126 potential participants, 26 did not meet the inclusion criteria, nine were not interested in the study, and 10 could not participate in the study for other reasons (failure to sign the informed consent form, lack of musical rhythm to participate in MMT). So, a total of 81 subjects were enrolled in this study. Figure 1 shows a flow chart of participants’ screening, enrollment, and randomization. Baseline characteristics of the randomized subjects were comparable across the three treatment groups (Table 1). There was no significant difference in baseline characteristics among the three groups.

**TABLE 1:** Baseline characteristics.

Characteristics	MMT group (*n* = 23)	ET group (*n* = 23)	Control group (*n* = 24)	*P* value
Age, mean (SD), years	64.090 (11.000)	65.650 (8.716)	61.580 (10.730)	0.391
Duration of illness, mean (SD), month	43.480 (11.390)	44.830 (9.316)	41.080 (10.260)	0.458
Women, No (%)	11 (47.826)	12 (52.174)	13 (54.167)	0.906
Height, mean (SD), meter	1.660 (0.067)	1.653 (0.067)	1.650 (0.085)	0.907
Hoehn & Yahr stage, No (%)				
2	5 (21.739)	3 (13.043)	5 (21.739)	
2.5	9 (39.130)	11 (47.826)	10 (43.478)	0.95
3	9 (39.130)	9 (39.130)	9 (39.130)	

MMT, music-based movement therapy; ET, exercise therapy; CON, control. The baseline characteristics of the three groups were comparable.

### Primary outcome

#### Gait cycle

**FIGURE 2:**
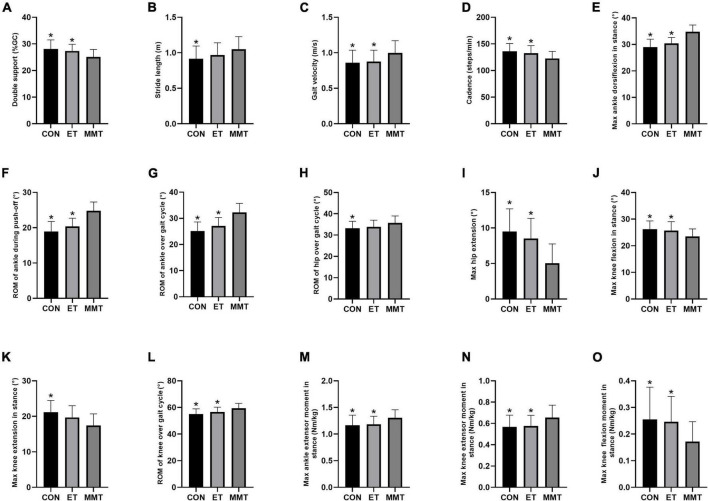
Gait parameters. Gait parameters were expressed in mean ± standard deviation. One-way ANOVA was used to compare the difference between CON, MMT, and ET. Significance level was set at 0.05. *p < 0.05, compared with the MMT group. ROM, range of motion; MMT, music-based movement therapy; ET, exercise therapy; CON, control. **(A)** Gait cycle, **(B–D)** kinematic parameters, **(E–L)** joint angle parameters and **(M–O)** kinetic parameters.

After 4 weeks of intervention, the double support time in the MMT group was lower than that in the control and ET groups (*F* = 6.647, *p* = 0.002). However, no significant difference was reported between the control group and ET group (*p* = 0.638) (Table 2; Figure 2A).

**TABLE 2:** Gait parameters.

Gait parameters	CON	ET	MMT	*F*	*P* value	*P* value (*post hoc*)		
						
						CON *vs.* ET	CON *vs.* MMT	ET *vs.* MMT
**Kinematic parameters**								
Stride length (m)	0.915 ± 0.181	0.967 ± 0.174	1.050 ± 0.178	3.481	0.037*	0.587	0.030*	0.248
Gait velocity (m/s)	0.860 ± 0.177	0.900 ± 0.159	0.997 ± 0.174	4.516	0.015*	0.942	0.021*	0.048*
Cadence (steps/min)	136.000 ± 14.63	132.700 ± 14.040	122.700 ± 13.240	5.877	0.005*	0.703	0.005*	0.042*
**Gait cycle**								
Double support (%GC)	28.120 ± 3.388	27.330 ± 2.561	25.120 ± 2.778	6.647	0.002*	0.638	0.002*	0.031*
**Joint angle parameters**								
Max ankle dorsiflexion in stance	28.960 ± 3.037	30.390 ± 2.231	34.750 ± 2.575	30.990	<0.001*	0.162	<0.001*	<0.001*
ROM of ankle during push-off	18.960 ± 2.804	20.390 ± 2.330	24.790 ± 2.502	33.530	<0.001*	0.145	<0.001*	<0.001*
ROM of ankle over gait cycle	25.130 ± 3.494	27.130 ± 3.195	32.290 ± 3.420	28.340	<0.001*	0.117	<0.001*	<0.001*
Max knee flexion in stance	26.170 ± 3.128	25.700 ± 3.350	23.500 ± 2.813	4.999	0.010*	0.860	0.012*	0.047*
Max knee extension in stance	21.130 ± 3.334	19.700 ± 3.295	17.420 ± 3.269	7.592	0.001*	0.309	0.001*	0.054
ROM of knee over gait cycle	55.130 ± 3.829	56.650 ± 3.563	59.420 ± 3.694	8.145	0.001*	0.349	0.001*	0.033*
Max hip extension	9.522 ± 3.175	8.522 ± 2.842	5.042 ± 2.710	15.400	<0.001*	0.479	<0.001*	<0.001*
ROM of hip over gait cycle	33.220 ± 3.275	33.910 ± 3.103	35.670 ± 3.319	3.594	0.033*	0.747	0.031*	0.159
**Kinetic parameters**								
Max ankle extensor moment in stance	1.165 ± 0.192	1.183 ± 0.152	1.303 ± 0.153	4.834	0.011*	0.923	0.016*	0.042*
Max knee extension moment in stance	0.568 ± 0.109	0.577 ± 0.099	0.654 ± 0.117	4.435	0.016*	0.960	0.023*	0.048*
Max knee flexion moment in stance	0.255 ± 0.122	0.247 ± 0.095	0.172 ± 0.075	5.094	0.009*	0.957	0.014*	0.030*

Gait parameters were expressed in mean ± standard deviation. One-way ANOVA was used to compare the difference between CON, MMT and ET. Significance level was set at 0.05. *Showed significant difference. ROM, range of motion; MMT, music-based movement therapy; ET, exercise therapy; CON, control.

#### Kinematic parameters

The cadence in the MMT group was lower than that in the control and ET groups (*F* = 5.877, *p* = 0.005). The gait velocity in the MMT group was higher than that in the control and ET groups (*F* = 4.516, *p* = 0.015). However, no significant difference was reported between the control and ET group (*p* > 0.05). The stride length in the MMT group was higher than that in the control group (*p* = 0.030). Nevertheless, there was no significant difference between the ET and MMT groups (*p* = 0.248) or the control group (*p* = 0.587) (Table 2; Figures 2B–D).

#### Joint angle parameters

For the ankle, the max dorsiflexion in stance (*F* = 30.990, *p* < 0.001), ROM during push-off (*F* = 33.530, *p* < 0.001), and ROM over gait cycle (*F* = 28.340, *p* < 0.001) in the MMT group were higher than that in the control and ET groups. However, no significant difference was reported between the control group and ET group (*p* > 0.05).

As for the knee, the max flexion in stance (*F* = 4.999, *p* = 0.010) in the MMT group was lower than that in the control and ET groups, and the ROM over gait cycle (*F* = 8.145, *p* = 0.001) in the MMT group was higher than that in the control and ET groups. However, no significant difference was reported between control group and ET group (*p* > 0.05). The max extension in stance in the MMT group was lower than that in the control group (*p* = 0.001). Nevertheless, there was no significant difference between the ET group and MMT group (*p* = 0.054) or the control group (*p* = 0.309).

Regarding the hip, the max hip extension (*F* = 15.400, *p* < 0.001) in the MMT group was lower than that in the control and ET groups. However, no significant difference was reported between the control group and ET group (*p* = 0.479). The ROM over the gait cycle in the MMT group was higher than that in the control group (*p* = 0.031). Nevertheless, there was no significant difference between the ET group and MMT group (*p* = 0.159) or the control group (*p* = 0.747) (Table 2; Figures 2E–L).

#### Kinetic parameters

After 4 weeks of intervention, the max extensor moment in stance (ankle: *F* = 4.834, *p* = 0.011; knee: *F* = 4.435, *p* = 0.016) in the MMT group was higher than that in the control and ET groups, and the flexion moment of knee in stance (*F* = 5.094, *p* = 0.009) in the MMT group was lower than that in the control and ET groups. However, no significant difference was reported between the control group and ET group (*p* > 0.05) (Table 2; Figures 2M–O).

### Secondary outcomes

#### Motor function

**FIGURE 3:**
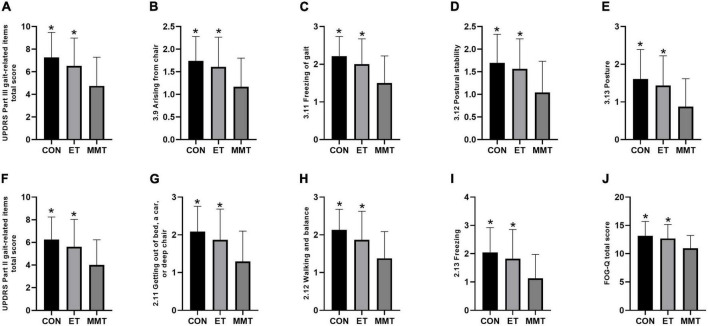
Change in FOG-Q and the comprehensive motor function. Secondary indicators were expressed in mean ± standard deviation. One-way ANOVA was used to compare the difference between CON, MMT, and ET. Significance level was set at 0.05. *p < 0.05, compared with the MMT group. MMT, music-based movement therapy; ET, exercise therapy; CON, control. UPDRS, Movement Disorder Society-Unified Parkinson’s Disease Rating Scale; FOG-Q, freezing of gait questionnaire. **(A–I)** Motor function; **(J)** FOG-Q.

The comprehensive motor function of patients was evaluated by UPDRS Part II and III gait-related items. After 4 weeks of intervention, the comprehensive motor function in the MMT group was lower than that in the control and ET groups (total score of UPDRS Part III gait-related items: *F* = 6.740, *p* = 0.002; arising from chair: *F* = 5.644, *p* = 0.005; freezing of gait: *F* = 7.665, *p* = 0.001; postural stability: *F* = 6.435, *p* = 0.003; posture: *F* = 5.852, *p* = 0.005; total score of MDS-UPDRS Part II gait-related items: *F* = 6.492, *p* = 0.003; getting out of bed, a car, or deep chair: *F* = 6.785, *p* = 0.002; walking and balance: *F* = 7.542, *p* = 0.001; freezing: *F* = 6.410, *p* = 0.003). However, no significant difference was reported between the control group and ET group (*p* > 0.05) (Table 3; Figures 3A–I).

**TABLE 3:** Change in FOG-Q and the comprehensive motor function.

Secondary indicators	CON	ET	MMT	*F*	*P* value	*P* value (*post hoc*)		
						
						CON *vs.* ET	CON *vs.* MMT	ET *vs.* MMT
UPDRS Part III gait-related items total score	7.261 ± 2.220	6.522 ± 2.466	4.750 ± 2.541	6.740	0.002*	0.556	0.002*	0.038*
3.9 Arising from chair	1.739 ± 0.541	1.609 ± 0.656	1.167 ± 0.637	5.644	0.005*	0.752	0.006*	0.042*
3.11 Freezing of gait	2.217 ± 0.518	2.000 ± 0.674	1.500 ± 0.722	7.665	0.001*	0.492	0.001*	0.027*
3.12 Postural stability	1.696 ± 0.635	1.565 ± 0.662	1.042 ± 0.690	6.435	0.003*	0.784	0.003*	0.023*
3.13 Posture	1.609 ± 0.783	1.435 ± 0.788	0.875 ± 0.741	5.852	0.005*	0.725	0.005*	0.040*
UPDRS Part II gait-related items total score	6.261 ± 1.982	5.609 ± 2.426	4.000 ± 2.226	6.492	0.003*	0.582	0.002*	0.041*
2.11 Getting out of bed, a car, or deep chair	2.087 ± 0.668	1.870 ± 0.815	1.292 ± 0.807	6.785	0.002*	0.604	0.002*	0.032*
2.12 Walking and balance	2.130 ± 0.548	1.870 ± 0.757	1.375 ± 0.711	7.542	0.001*	0.398	0.001*	0.039*
2.13 Freezing	2.043 ± 0.878	1.826 ± 1.029	1.125 ± 0.850	6.410	0.003*	0.704	0.003*	0.030*
FOG-Q total score	13.170 ± 2.534	12.700 ± 2.458	10.960 ± 2.274	5.474	0.006*	0.782	0.007*	0.043*

Secondary indicators were expressed in mean ± standard deviation. One-way ANOVA was used to compare the difference between CON, MMT and ET. Significance level was set at 0.05. *Showed significant difference. MMT, music-based movement therapy; ET, exercise therapy; CON, control. UPDRS, Movement Disorder Society-Unified Parkinson’s Disease Rating Scale; FOG-Q, Freezing of Gait Questionnaire.

### FOG-Questionnaire

The FOG-Q (*F* = 5.474, *p* = 0.006) in the MMT group was lower than that in the control and ET groups. Nevertheless, no significant difference was reported between the control group and ET group (*p* = 0.782) (Table 3; Figure 3J).

## Discussion

This study evaluated the efficacy of MMT in improving gait disorders, including FOG, which severely reduced the QOL of patients with PD. Results demonstrated the improvements in gait disorders as measured by gait analysis, UPDRS Part II, and Part III and FOG-Q. The key gait parameters findings in this study showed that MMT could significantly reduce the double support time and improve the joint angle, kinematic, and kinetic parameters.

For patients with PD, double support time reflects dynamic postural stability (Fasano et al., 2016). Increased double support time, a significant injury risk, may be an indicator of instability that had a significant impact on the gait pattern in PD patients with FOG (Zanardi et al., 2021). The preservation of dynamic stability in PD with FOG was accomplished by postural synergies (He et al., 2018). The extension of double support time during the gait cycle offers a longer time to achieve gait stability, thereby diminishing the requirements for the postural control system (LaPointe et al., 2010; Williams and Martin, 2019). Furthermore, participants surmount the dread of falling by boosting double support time to restore gait stability (Michalitsis et al., 2019). Thus, we concluded that MMT might increase gait stability in PD with FOG. Aside from the double support time, the kinematic parameters were an index of gait instability and fall risk (Thompson et al., 2017; Peppe et al., 2019). The kinematic features of gait in PD with FOG were characterized by slower velocity, shorter stride length, and higher cadence. PD with FOG undergoing MMT showed a notably slower cadence, longer stride length, and faster velocity. Increased stride length and slower cadence were instrumental in PD with FOG for that they perhaps were conducive to avert the emergence of sequence effect, thereupon then contributing to ameliorating FOG. Significantly, lower ROM values of knee and ankle were found in patients compared to controls. In comparison, MMT prominently expanded joint angle offset. Research also indicated that gait disorder in PD with FOG derived from a discrepancy between cortically selected motion amplitude and the authentic proportion of leg movements implemented during walking (Morris et al., 2005; Miranda-Domínguez et al., 2020). MMT might enable participants to focus on walking tasks and mobilize sensitized motion control strategies which circumvented the damaged basal ganglia, therefore, giving rise to the legs’ flexion-extension flexibility. The enhancement of lower limbs joints movement perhaps be the premise of the stride length lengthening (Struzik et al., 2016). Factors, for instance, the betterment of ROM at the ankle joint in the time of push-off, maximal hip, and knee extension in stance phase, presumably were conducive to lengthening the stride length. With the improvement of dynamics, the parameters of gait kinematics were also improved. Significant kinetic amelioration in the joints of lower extremities was also found in the MMT group. In addition, the tarsoptosis gait in PD with FOG perhaps was bound up with a lessened heel rocker, deficient knee extension, or insufficient hip flexion.

In spite of the promising effects of medical and surgical management, sequela of the movement disorders are not prevented and disabilities owing to mobility incommodiousness remain (Schootemeijer et al., 2020). Many gait defects in patients with PD, such as FOG, are relatively insensitive to drugs and surgical remedies (Ferrazzoli et al., 2016). Rehabilitation programs were important adjuncts to medical interventions that address residual motor deficits through task-oriented exercises. Exercise training was a significant therapeutic means in the rehabilitation setup. A prevalent and potential strategy to ameliorate gait performance and mobility is to apply external cue technology, especially rhythmic music (Harrison et al., 2018). Music probably touches off physiological enjoyment associated with reward and emotions, which probably draw attention from sensations for instance fatigue in turn and enhance therapy compliance over a long period of time (Jomori et al., 2013; Farbood et al., 2015). These active roles of music perhaps indicate the potentiality of incorporating music into exercise therapy in the management of PD. MMT, an integral part of cognitive assignment training, entails practitioners to remembering the name of each action as well as imitating the action. Ultimately, participants are required to complete a range of actions integrated with music, which ameliorate the cognitive functions of PD, such as visuospatial ability and executive ability. Thus, 4-week MMT was able to improve the gait performance of PD.

Various mechanisms have been proposed to explain the therapeutic effect of MMT on FOG in PD. One potential mechanism involves the alteration of neural pathways. Impaired basal ganglia function in patients with PD could impact their perception of rhythm, subsequently leading to gait freezing (Jones and Jahanshahi, 2014). This could be compensated by the complement of the cerebellar network, which is usually spared in PD. In addition, the activation of the cerebellar network is dependent on external (e.g., auditory or visual) stimuli (Ashoori et al., 2015). Another potential mechanism involves rhythmic entrainment, which synchronized the auditory system with the motor system to facilitate movement (Thaut et al., 2014). In addition, the auditory environment and motivational effects of music might accelerate motor learning (Schaefer, 2014). Music might modulate the activity of specific brain regions by promoting the production of brain-derived neurotrophic factors (Clements-Cortes and Bartel, 2018).

In summary, PD with FOG was inclined to present with gait disturbance, including extended biphasic support time, shortened stride length, slower velocity, and shrunken ROM in lower extremities joints. These gait deficiencies gradually worsen along with the progression of disease, greatly reducing the quality of life. The present research revealed that abnormal gait characteristics in the MMT group were significantly improved. Thus, MMT can be used as a physical therapy method, which was conducive to the improvement of FOG in PD. Nevertheless, electromyography (EMG) activity in lower extremities was not investigated in the presented study. Future research is suggested to explore the effect of MMT on EMG of related muscle groups and its impacts on muscle power generation. Furthermore, the results did not provide clear evidence that whether the benefits of MMT will fade over time. Additionally, it is imperative to expand the sample size and carry out longer-term interventions and follow-up.

## Conclusion

Music-based movement therapy improved gait disorders in PD patients with FOG, thereby improving their comprehensive motor function.

## Data availability statement

The datasets used and/or analyzed during the current study are available from the corresponding author on reasonable request.

## Ethics statement

The studies involving human participants were reviewed and approved by Ethics Committee of Shanghai Second Rehabilitation Hospital. The patients/participants provided their written informed consent to participate in this study.

## Author contributions

K-PL, Z-QZ, and J-GX equally contributed to the design or implementation of the research. B-HS and X-HW were in charge of data collection. J-QS was responsible for data analysis and interpretation. Z-QZ, K-PL, and Z-LZ assisted in drafting the manuscript, which was critically revised by J-GX. All authors approved the final manuscript.
